# Artificial Intelligence-Assisted Colposcopy: Deep Learning Multi-Class Segmentation of Anatomical Structures and Pathological Findings for Cervical Cancer Screening

**DOI:** 10.3390/cancers18091485

**Published:** 2026-05-05

**Authors:** Marcin Jurczak, Łukasz Charzewski, Beata Goźlińska, Paweł Albrycht, Kacper Kobus, Artur Ludwin, Zoulikha Jabiry-Zieniewicz, Sylwester Kominek, Grzegorz Basiński, Bartosz Korzeb, Barbara Ewa Suchońska

**Affiliations:** 1 Proacta SA, Srebrna 16, 00-810 Warsaw, Poland; marcin.jurczak@proacta.pl (M.J.); lukasz.charzewski@proacta.pl (Ł.C.); beata.gozlinska@proacta.pl (B.G.); pawel.albrycht@proacta.pl (P.A.); kacper.kobus@proacta.pl (K.K.); 2 University Center for Women’s and Newborn’s Health, Medical University of Warsaw, 1/3 Starynkiewicza Sq, 02-015 Warsaw, Poland; artur.ludwin@wum.edu.pl (A.L.); zoulikha.jabiry-zieniewicz@wum.edu.pl (Z.J.-Z.); skominek@uczkin.pl (S.K.); gbasinski@uczkin.pl (G.B.); bartosz.korzeb@wum.edu.pl (B.K.); 3 1st Department of Obstetrics and Gynaecology, Medical University of Warsaw, 1/3 Starynkiewicza Sq, 02-015 Warsaw, Poland

**Keywords:** colposcopy, precancer screening, semantic segmentation, deep learning, computer vision, AI

## Abstract

**Background**: Accurate colposcopy assessment is essential for detection of cervical precancerous lesions. However, the diagnostic performance depends heavily on the examiner’s experience and subjective interpretation. Recent advances in artificial intelligence offer new opportunities for automated image analysis. In particular, deep learning models have shown promise for colposcopy image analysis intended for precancer screening. However, their limited interpretability restricts their translational value in clinical gynecology. This study leverages a custom dataset of expert-annotated digital colposcopic images to quantify the diagnostic strengths of these architectures. **Methods**: A comparative analysis was provided of the well-established convolutional neural network YOLOv11 and the modern transformer-based RF-DETR architecture, both of which were trained for the segmentation of 10 distinct classes. Target objects included anatomical structures, medical instruments and colposcopic findings. **Results**: Our results demonstrate that the YOLO architecture provides better performance for anatomical structures, whereas the RF-DERT reaches higher scores for colposcopy findings. These findings demonstrate transformer architecture superiority in more nuanced segmentation of clinically relevant findings, providing an interesting framework for computer-aided decision support systems in colposcopy. **Conclusions**: Both YOLOv11 and RF-DETR enable effective segmentation of colposcopic images, with performance dependent on class size and characteristics. Best results were achieved for large anatomical structures, while small and underrepresented findings remained challenging due to class imbalance. YOLO offers greater stability and efficiency, whereas RF-DETR performs better on more complex cases. Limitations include data imbalance, variable image quality, and annotation inconsistencies; future work should address these issues to improve generalization.

## Simple Summary

This study addresses the challenge of accurately detecting the early signs of cervical cancer during colposcopy, a procedure whose effectiveness is often dependent on the clinician’s level of experience. The authors evaluate whether modern artificial intelligence methods can support this process by improving the analysis of medical images. Using a dataset of colposcopy images annotated by experts, the authors compare two advanced approaches for identifying important features, such as anatomical structures and abnormal findings. The results demonstrate that different methods excel in different tasks, with architecture playing a key role in detecting subtle, clinically significant changes. These results imply that AI could be a valuable support tool for clinicians, potentially improving diagnostic accuracy, enabling earlier detection, and leading to better patient outcomes.

## 1. Introduction

Cervical cancer is the fourth most common cancerous disease among women. Worldwide, over 500,000 cases of cervical cancer are diagnosed annually, of which over 300,000 cases lead to patient death [1]. Almost all cases of cervical cancer are associated with human papillomavirus (HPV) infection. The vast majority of these cases are caused by infection with types 16 and 18 [2]. Other risk factors, such as smoking, the long-term use of oral contraceptives and immunodeficiency (including HIV infection), also play a significant role in the development of cancer [3]. Studies suggest that HPV vaccination between the ages of 9 and 12 might prevent over 90% of cervical precancer and cancer cases to the point where cervical cancer would no longer be a public health concern [4].

In the modern screening model based on HR HPV testing, molecular testing serves as primary screening, while cytology is used as a triage (secondary) test to further stratify risk in women with positive HPV results [5]. Colposcopy is not a component of screening but rather a step in diagnostic work-up, serving as a bridge between a positive screening test result and histopathological verification. By using a magnifying optical instrument combined with chemical markers, a colposcopist can identify abnormal epithelial patterns visually and potentially perform a biopsy [6].

Currently, digital colposcopy is an essential procedure for cervical intraepithelial neoplasia (CIN) assessment, which is divided into three grades. Although the LSIL/HSIL (Low-grade Squamous Intraepithelial Lesion/High-grade Squamous Intraepithelial Lesion) terminology is currently recommended by the WHO and the Lower Anogenital Squamous Terminology (LAST) guidelines, the CIN classification remains commonly used in histopathological reporting and clinical communication. For clarity and consistency with the provided clinical metadata, this manuscript uses CIN terminology. In general, CIN1 corresponds to LSIL, whereas CIN2 and CIN3 are classified as HSIL. CIN1, covering the basal third of the epithelial thickness, is a potentially reversible effect of HPV infection and is characterized by a high rate of spontaneous regression. Although CIN2 covers two-thirds of the epithelial thickness and still has the potential to regress, it is associated with a higher risk of progression. Depending on the patient’s age and history, it may be treated as a high-grade lesion. CIN3, which occupies more than two-thirds or even the entirety of the epithelium, is a direct precursor to invasive squamous cell carcinoma, and intervention is required to prevent malignancy [7].

The standard colposcopy protocol involves a visual examination of the vulva and vagina, followed by an initial assessment of the cervix at 4–15× magnification. The cervix is then carefully cleansed with saline and re-evaluated using a green filter. Then, 3–5% acetic acid is applied, after which the cervix is examined after one and, optionally, three minutes. Once lesions have whitened in response to the acid, they can be selected for targeted biopsy [8]. However, the standard colposcopy protocol does not include an additional staining step, namely the Schiller test, which uses Lugol’s solution to visualize and differentiate abnormalities more effectively. The Schiller test causes a dark, mahogany color to appear on the glycogen-containing physiological squamous epithelium, while squamous intraepithelial lesion (SIL) cells, which lack glycogen, remain unstained [9].

The accuracy of colposcopy depends largely on the experience of the person performing the examination [10]. Interpretation is subjective, and there are well-documented differences between colposcopists. Results also vary depending on diagnostic thresholds. In clinical practice, colposcopy results should be considered in conjunction with cytology and HPV test results. Consistent results reduce the risk of misclassification [11,12]. Considering that all colposcopic findings are visible and visual observations are documented, it is possible to apply computer vision methods for results analysis. Although the static nature of such analysis does not allow for observing the temporal dynamics of acetowhitening, all images in this study were acquired following a rigid clinical protocol to ensure high reproducibility and consistency of the documented features. In this study, we present our approach for developing a computer-aided tool for processing colposcopy data, starting from data collection and annotation, through deep-learning methods, for the prototype of a clinical application intended for future integration into the patient examination process.

Recent years in diagnostic imaging have seen a growing interest in computer-aided analysis, which is promising for colposcopic image analysis. There are two main approaches in studies attempting to apply computer-aided diagnosis in colposcopy. First is to use traditional machine learning methods with supervised learning, which involves the independent extraction of image features that would be searched for in a collection of images. Second is the utilization of solutions based on deep-learning—neural networks, which independently search for recurring patterns in a dataset. Deep learning models are characterized by their unique ability to precisely extract and classify features. However, the computational complexity of these algorithms is much greater than that of classical machine learning algorithms, which directly translates into greater development challenges and increased computational costs [13,14].

The classic approach to data analysis is faster and requires smaller datasets. Its undoubted advantage is the transparency of the result and interpretability—the ability to explain the model’s decisions [15,16]. However, it can be ineffective for tasks requiring the classification of many features or when dealing with unstructured data. For this reason, they are considered superior for processing tabular data, but they underperform when analyzing other modalities, such as sounds or images [15,16].

The use of neural networks addresses this problem—the algorithm itself learns to find patterns in unsystematized data in many applications, demonstrating remarkable performance. However, deep model training is computationally expensive. It also requires significantly more data to obtain satisfactory results. An additional problem is the ambiguity of decisions made by the model, which acts as a “black box”—the classification result is known, but it is not clear which features of the set influenced the model’s decision [17].

One of the well-suited deep model architectures for interpretable image analysis is the convolutional neural network (CNN). Its design utilizes so-called convolutional layers, which automatically optimize feature extraction on increasingly complex levels, leading to the creation of feature maps without prior feature engineering [18]. This makes the CNN approach relatively flexible, which is particularly important for heterogeneous datasets [13].

A crucial stage in research using convolutional neural networks is evaluating the quality of the model’s results. Most commonly used evaluation metrics include

- Sensitivity (recall)—measures how many true positive cases have actually been detected; this is a key evaluation metric when the omission of a positive result is particularly severe for the subject under study [19].
- Precision—describes the fraction of predictions that were detected correctly [19].
- IoU (intersection over union)—calculates the ratio of the common area between two regions of interest to their union. Higher IoU values indicate a better spatial fit between the detected region and the ground truth [20].
- Dice (Dice–Sørensen coefficient)—measures similarity between two areas similarly to IoU but enhances the impact of the common area [21].
- F1-score—also known as F-measure, has the form of a harmonic mean between precision and recall. The interpretation of the F1-score is that a higher F1-score indicates better segmentation algorithm performance [22].
- mAP (mean Average Precision)—is the mean of average precision (AP) across all classes, which is a measure of the trade-off between recall and precision. To compute this metric, a precision–recall curve is calculated (precision on the y-axis, recall on the x-axis). The area under the curve is the mean of precisions in the set of recall points [22].

One broadly used CNN architecture for image object detection and segmentation is the YOLO (You Only Look Once) algorithm [23], which is valued in diagnostic imaging for its speed and ability to be used in real time, for example, for camera images [24]. The YOLO algorithm is constantly being developed; hence, it has different versions, optimized for speed and specific applications [24]. In recent years, YOLO has been successfully used at various stages of colposcopy image classification [13,19,20].

The YOLOv11 model offers solutions that improve its ability to detect objects, segment them, and estimate their exact position with pixel-level accuracy [25]. The latter feature is particularly valuable when analyzing medical images, especially in tasks such as detecting tumors or organ boundaries [25].

YOLOv3 performance has been evaluated for initial cervical detection in colposcopic images [13]. The study dataset consisted of sets of colposcopic images from 1180 patient visits performed with a pocket colposcope. The training set contained 756 images after acetic acid wash, while the test set contained 124 images after acetic acid wash, both sets randomly sampled with the proviso that the results of the same patient were not divided into two different sets [26].

The detection stage using YOLOv3 allowed the authors of the experiment to remove features that were clinically irrelevant from the perspective of cervical cancer diagnosis (vaginal walls, speculum) from the set. This operation also reduced the size of the images going to the target task, i.e., the direct detection of cervical cancer, performed with an accuracy of 0.997 on the test set [13].

A successor version, YOLOv5, was used to isolate the cervix in colposcopic images to remove disturbances (vaginal walls, sticks, speculum) that could negatively affect the subsequent classification of transformation zones of the cervix [19]. The authors emphasize the precision and speed of YOLOv5 in marking image segments, thanks to which the images were cropped to an area of interest measuring 224 × 224 pixels for final classification [19]. After isolating the cervix from the colposcopy image, the authors use another CNN model, MobileNetV3, to classify the type of the Transformation zone, which is the area where stratified squamous epithelium develops. In this area, precancerous changes occur most frequently. The final model achieved 83.97% classification accuracy [19].

Depending on the approach, multiple versions of YOLO can be used at different stages of the experiment [20]. The authors of the study compiled a set of images focusing only on the stage of the examination after application of acetic acid. Their set contained data from 720 patients, of whom 418 had a diagnosis of abnormal cervix, and the remaining 302 constituted the control group. In the first stage of colposcopy image analysis, which was to isolate the cervix from the images, YOLO Fastest was used to balance detection accuracy and very fast processing time. The resulting model can detect the cervix area with an accuracy of 99.49% and sensitivity of 99.49% [20]. The input data for the second stage of the study were the segments of images extracted from the previous stage containing a view of the cervix. This stage of the study aims to identify images containing cervical precancerous lesions. The current model supports only two classes—images that have passed the VIA test (visual inspection using acetic acid [27]) and images that have failed the VIA test. The problem was addressed using the YOLOv8-seg model [28], which is a very efficient model for real-time segmentation with significantly higher speed and accuracy in object detection tasks than previous versions of YOLO [20]. As a result, the authors achieved 73% mAP score in segmenting the location of cancerous lesions and 40% IoU score in accurately isolating them from the rest of the organ visually [27].

The latest version of YOLO is YOLO26, which, according to its creators, is faster, more efficient, and easier to integrate than previous versions. It is also to be optimized for segmentation and accurate positioning tasks [26]. Version YOLO26 introduced a new feature compared to earlier versions of YOLO models: oriented detection. This means it detects rotated objects and recognizes their orientation angle to improve accuracy [29].

The use of transformer architectures has also become a common approach in image analysis. In recent years, they have been growing in popularity in the medical field due to their precision. They are based on the verification of the global context of an image, which makes them a relevant tool for recognizing even microscopic alterations in medical images. In contrast to CNN, transformers are less data efficient but have a higher capacity, which, as the availability of the data grows, becomes more important over time [30].

A recent study aimed to verify the effectiveness of transformer architecture in the automatic colposcopy image classification using a dedicated LSTEDL-CCS model [31]. The analysis process begins with preliminary image processing using a Wiener filter, followed by feature extraction from the processed image using a Swin transformer designed to effectively capture image variables. The final and key element of LSTEDL-CCS is the ensemble learning method, which combines Autodecoder, BiGRU, and the Deep Belief Network. The application of ensemble modeling allows combining different models to improve predictions and collectively interpret complex patterns in the provided data. The model was trained and validated via an open-source Kaggle Dataset containing 600 colposcopic images with three classes representing types of cervix transformation zone [32]. The obtained accuracy, precision, sensitivity, and specificity of the proposed model ensemble were 99.44%, 99.22%, 99.05%, and 99.57%, respectively, which were all higher than any other compared models, including TernausNet-DLV2, InceptionV3, and EfficienNet-B3 [31].

Another interesting model, SegFormer, presented in 2021, combines a hierarchical transformer encoder with a lightweight multilayer perceptron. It was designed to overcome the limitations of traditional classification and standard vision transformers and leverage the global context of the provided data. SegFormer architecture presents strong potential in the classification [33] and segmentation [34] of medical images. The use of SegFormer for the segmentation of colposcopy images after acetic acid washing has been tested on a dataset from a hospital in Palembang, Indonesia [34]. A multi-label segmentation structure recognizing not only precancerous lesions but also anatomical elements such as epithelium and external os has been trained. Ultimately, five precancerous/anatomical classes were identified: cervical area, external os, columnar area, precancerous lesion, and transformation zone. In order to improve effectiveness with a relatively small database of colposcopy (769 images after acetic acid washing), the researchers developed a lesion-specific augmentation data enhancement method. By employing techniques such as brightness modulation, angle modulation, and image Gaussian noise artifact injection, they made the system highly resistant to low-quality images. As a result, their SegFormer model achieved an overall performance with a balanced accuracy of 95.09% and a sensitivity of 92.64%. The methods utilized in this study, in combination with SegFormer, are very promising in diagnosis. However, the authors have not implemented this solution in clinical practice and point out that further research is required [34].

An interesting technological development is RF-DETR, a relatively light-weight vision transformer [35]. Although currently still under development, it is actively being explored for use in the detection of cancerous changes in medicine [36]. At present, no research team has used it to identify structures in colposcopic images, but there are promising results using RF-DETR to identify abnormal changes in cervical histopathology [36]. The potential of RF-DETR has been confirmed in a study on precision urine microscopy for renal and systemic disease diagnosis [37]. The study compared the diagnostic capabilities of standard parameters such as color and pH with those of automatic identification of morphological elements in sediment. RF-DETR with integrated DINOv2, a self-supervised vision transformer working as a visual backbone [38] and deformable attention, drastically outperforms existing diagnostic systems based on YOLOv8 or Faster R-CNN [39] in terms of efficiency and speed of analysis [37].

## 2. Materials and Methods

To ensure peak performance, which is required in medical diagnosis, such a system must be based on a model trained on extensive datasets consisting of precisely tagged and masked images. Achieving the high clinical reliability required in medical diagnostics necessitates a large volume of data, the involvement of experienced experts in the field, and the use of rigorously annotated, double-checked datasets [40]. Therefore, for this study, we have utilized our in-house collected and carefully annotated colposcopy dataset.

### 2.1. Data Acquisition and Standardization

The study utilized high resolution digital colposcopic images acquired under standardized clinical conditions at the University Center for Women’s and Newborn’s Health of the Medical University of Warsaw (UCZKiN), adhering to the technical and procedural requirements defined by the Polish Society of Colposcopy and Cervical Pathophysiology [8]. All examinations followed a multistep protocol, including macroscopic inspection, saline preparation, and the application of 3–5% acetic acid. Images were collected using colposcopes providing the required 4–15× magnification range, ensuring adequate visualization of the Transformation zone (TZ) and squamo-columnar junction (SCJ), which are strategic landmarks for a diagnostic colposcopy. Data collection spanned a period during which the clinical equipment was upgraded, resulting in a dataset composed of images from two different colposcopic systems. To ensure data consistency, and minimize variability in the neural network training set, images were captured at fixed intervals after chemical marker application.

The dataset predominantly consists of images from two primary colposcopic systems, forming two main categories based on their resolutions: 7637 images with a resolution of 717 × 539 px, acquired using the older MI24K/S system (manufactured in 2012, Warsaw, Poland), a device equipped with a column mount, video track, and Miniiris archiving software (version 1.5.7.2010-01-24), providing magnification up to 5× (optical) and 25× (eyepiece), and, following the equipment upgrade, an additional 2440 images, captured at a higher resolution of 1280 × 960 px using the LEISEGANG Model 3MVC LED USB system (manufactured in 2020, Berlin, Germany). This newer device features a 300 mm working distance, optical magnification up to 30×, and high intensity LED illumination ranging from 45,000 to 52,000 lx, with an integrated 1.3 MP camera operated via VCK27 Viewer software (version V1.7.5). To maintain high data consistency for the neural network training set, a filtering process was implemented to account for variations in image dimensions encountered during clinical practice. Apart from two main categories, the dataset also contained a subset of 120 additional images with resolutions ranging from a minimum of 167 × 130 px, to a maximum of 2151 × 1615 px.

### 2.2. Dataset Preparation and Annotation

The initial phase of the study involved organization of the dataset and the establishment of a standardized annotation environment. First, all patients’ data imprinted on the images were covered with a black rectangle to ensure anonymity and prevent data leakage in further model training. A total of 10,184 high-resolution images were uploaded to Computer Vision Annotation Tool (CVAT) [41], version 2.37.0, deployed on-premise, an open source platform selected as an easy-to-use, collaborative pixel-wise segmentation tool.

The annotators' team consisted of three medical students belonging to a student scientific society, four OBGYN residents, and a panel of eight gynecologists specializing in colposcopy. The workload was distributed among the team members to maximize both speed and accuracy of data annotation. Standardized labeling guidelines, tags and masks within CVAT were created to take care of the consistency across all annotators. A printable checklist containing a list of classes to annotate was created and distributed. Images were partitioned into subsets and assigned to particular annotators based on their level of expertise.

The annotation process followed a multi-stage verification procedure. Initial annotations for each examination were made by medical students, who focused primarily on anatomical structures. These were subsequently checked and corrected by residents or specialists, who were responsible for identifying colposcopic findings and performing cross-checks. To ensure maximum data integrity and consistency, the final verification of all annotated images was performed by a single supervisor—a senior expert in colposcopy—who monitored the entire process.

### 2.3. Classification Tags and Segmentation Masks

In order to create a comprehensive dataset, two distinct types of annotations were prepared: categorical tags and pixel-level segmentation masks. Each colposcopic image was assigned a set of tags to mark its overall clinical status, technical parameters, and procedural context. The labels were organized into four main categories, as shown in Table 1. These labels allowed for further image filtering and assessment.

To annotate objects’ spatial localization, experts used a brush tool in CVAT to mask the characteristic area of anatomical structures and findings. To refine the clinical descriptions, specific attributes were added to some classes, such as distinguishing between the Original squamous epithelium and the Metaplastic squamous epithelium. To our knowledge, this is the first colposcopy dataset providing such a level of image characteristics. Conveniently, the hierarchical labels organization allows for combining or separating particular classes in experiments. In addition, masking of obstacles such as Medical instruments, Blood, or Mucus was performed. The number of each class object’s occurrences is presented in Table 1. To date, 6263 images of the total dataset have been annotated and evaluated. These masks provide the ground truth for segmentation, allowing the model to distinguish between healthy tissue, lesions, and environmental artifacts.

### 2.4. Quality Control and Annotation Challenges

During the study, several challenges concerning quality in the labeling process were encountered, for example, leaving the default Primary squamous epithelium attribute instead of Secondary squamous epithelium. These errors often stemmed from the repetitive nature of labeling features. To ensure high annotation reliability, the process was supervised by a domain expert, with inconsistencies corrected during dataset preparation. Additional quality reviews during model preparation identified and resolved a small number of errors: 24 instances of metaplastic squamous epithelium mislabeled as original, 3 cases of erythroplakia, 1 polyp, and 8 medical instruments. All identified inconsistencies were corrected prior to final model training.

However, given the large volume of data and high diversity of classes, detecting every individual mistake remains a challenge. It must therefore be acknowledged that despite rigorous elimination efforts, a marginal number of annotation errors may persist in the final dataset. This variability is a factor that must be considered during the final training and validation of the neural network models.

The research was based on colposcopic images and annotations made by specialists in the CVAT system. The data came from typical clinical practice, and a detailed description of the data and preparation details is provided in Section 2.1, Section 2.2 and Section 2.3. For the purposes of training selected models, segmentation masks assigned to anatomical structures and findings were used, together with additional attributes, e.g., type of Squamous epithelium. The annotations were exported from the CVAT system, which enabled automatic conversion to COCO 1.0 format, used in transformer-based model training, and CVAT for Images 1.1 (XML) format, used in CNN-based model training [41].

The exported data was processed using a script that adjusted it to the model requirements. The script parsed an XML file with annotations, collecting information about the resolution, stage, available labels, and attributes for each image. The data were then filtered based on labels and tags corresponding to specific stages of the study. Images marked as unusable and low-quality photos were rejected. In order to limit the number of resolution variants, an automatic selection of the two most common was introduced. This allowed us to avoid problems with scaling images of significantly different sizes. Images that did not have any annotations were eliminated from further processing.

An important element of the processing pipeline involved handling Run-Length Encoding (RLE) masks exported from CVAT. The script allowed RLE to be decoded into a binary mask and then converted into a set of contours. The contours were simplified; those that were too small, below 30 pixels, were discarded, and the rest were converted to the format required by the model architecture. Patient age and menopausal status were not used as input variables for the model.

A significant difficulty was encountered during the analysis of Squamous epithelium, which often occurs in the form of a ring structure with an empty area inside, forming a so-called donut-shape [42]. The standard representation of polygons. storing the outer and inner contours as a single contour, caused problems visible in the form of artifacts occurring in the model predictions. To address this issue, the processing pipeline was extended to handle the donut-shape problem. Contours were detected using hierarchical information and then merged into a single polygon using a connector line of one-point width between the nearest points of the contours. The polygons prepared in this way were scaled to the range of 0 to 1, relative to the width and height of the image, and then saved in label files as (x, y) sequences.

Another problem was areas marked with the same label but consisting of many disconnected components. In such cases, the components were divided into separate instances, and each was treated as an independent polygon to avoid an artificial connection of distant fragments of the same anatomical structure or finding.

After filtering and mask preparation, each dataset was divided into subsets: training, validation, and testing in 70/20/10 proportions. The decision to use an image-level split was primarily motivated by the multi-step nature of the colposcopy protocol. Each procedural stage induces significant and distinct visual changes in the cervical epithelium, effectively presenting the model with different diagnostic features. To rigorously address the risk of data leakage, we performed a secondary validation using a patient-level split. This analysis confirmed the robustness of our approach, as performance metrics remained stable for more numerous classes. This suggests that the models generalized to pathological patterns rather than individual patient characteristics.

Additionally, two training variants were introduced—extended and general. In the general variant, the label is defined as a whole class, without distinguishing attributes (e.g., Squamous epithelium). In the extended variant, the label is broken down into subclasses according to its attributes (e.g., Metaplastic squamous epithelium). In both variants, additional images not containing any instances of the considered class were allowed to be added as background examples. For each considered class, a specific number of background images were selected in which the given class does not appear. In the extended variant, a distinction was also made between difficult backgrounds, which were annotated with a different attribute of the analyzed class, and general backgrounds, where the class instance is not present at all. This approach allowed us to control the complexity of the data, which is particularly important in the case of discrimination between similar classes.

The study used two main models for segmentation: a CNN-based model, YOLO v11 (version 11n), and based on the transformer architecture RF-DETR (RFDETRSegPreview). This choice was motivated by the need to compare CNN-based models widely used in colposcopy with modern transformer models. The packages used for modeling in the YOLO variant are Ultralytics 8.3.204 (PyTorch 2.8.0+cu128) and, in the case of RF-DETR, Rfdetr 1.3.0 (PyTorch 2.2.2, Roboflow 1.2.11). YOLO is a well-researched model, widely used in many fields, which makes it a solid reference point. RF-DETR is a newer model and offers a different approach to image segmentation through the use of a transformer, which is an interesting alternative to explore and compare. Both models were trained on the same data subsets, allowing for a comparison of their performance on the identical colposcopy data. A comparison of the most important features of both models is summarized in Table A1 (Appendix A).

The selection of training hyperparameters for the YOLO model was preceded by a series of optimization experiments using the Optuna package 4.6.0 [43]. For selected classes (including Squamous epithelium, Cervix, External os, Polyp, Erythroplakia, Medical instruments), the hyperparameter space was automatically searched, including the number of epochs, batch size, choice of optimizer (AdamW/SGD) and its parameters, augmentation intensity and type, and background image contribution. Various versions of the pretrained YOLO v11 model on the COCO dataset (n, s, m, l) were also tested [44]. In experiments with Optuna, an objective function based on YOLO segmentation results was optimized—the value of the author’s combined indicator for masks given by 0.7 × F1 + 0.3 × mAP50-95. This indicator was determined experimentally, taking into account simultaneous consideration of both segmentation performance (F1) and quality at different overlapping thresholds (mAP50-95). Combining multiple metrics into a single indicator is commonly used practice while training and tuning YOLO models [45]. Analysis of the best attempts showed that the gains from further parameter optimization are small compared to the computational costs. However, three segmentation training configurations of pre-trained YOLO v11n-seg models clearly outperformed the others, repeatedly providing the best results, and these were selected as the variants used for further experiments. The most essential information about chosen models was presented in Table A2, Appendix A.

The models configurations differ in their approach to augmentation and training, but they all use the YOLO v11 model in the nano-seg variant, an input resolution of 640 pixels, and a batch size selected by default according to GPU capabilities.

Configuration 1 used the AdamW optimizer with a small learning rate, a maximum of 200 training epochs, and a set of mild geometric and color transformations (small rotations, scaling, translations, color and saturation modifications, no mosaic or mixup). Configuration 2 used longer training (up to 300 epochs), more intense transformations (greater range of rotations, scaling, and translations), and the use of mosaic, mixup, and copy–paste, which was aimed at increasing data variety in classes with limited number of instances. Configuration 3 was two stages: initially, the first layers of the model were frozen (transfer of learned features from training on general sets), and only the higher layers of the network were trained on a small number of epochs; then, the entire model was unfrozen and the full fine-tuning was performed with moderate augmentation to avoid overfitting on small sets.

The proper experiments were conducted by training a separate segmentation model for each class in the general variant, i.e., without splitting the label into subclasses according to its attributes. The exception was Squamous epithelium, for which an extended variant was used, distinguishing between separate models for Original and Metaplastic epithelium. The selected strategy of training one model per class, simplifies the interpretation of results, limits the interaction between classes (number of instances, imbalance) and helps to maintain individual models with the increasing number of annotations. The research also included training of multiclass models, but the results were significantly worse than in the case of the selected strategy. Although this strategy is less commonly used in practice, the results from individual models can be combined into a final segmentation map without overestimating the results because they are evaluated independently using pixel-level segmentation metrics.

The RF-DETR model was trained on identical data splits (training, validation, and test sets) using the same learning strategy as for YOLO—one model per class. In this case, COCO-format annotations prepared in earlier stages were used. For RF-DETR, a single unified output configuration was adopted for each training by setting the most important parameters in agreement with recommendations of the model developers and our own experiments conducted on colposcopy data. The key model and training parameters were presented in Table A3, Appendix A.

The training was performed in 350 epochs using EarlyStopping with a patience of 15 epochs. A small batch size (2) was used, which was effectively increased to 8 thanks to gradient accumulation. A fixed learning rate of 0.0001 was used. The number of object queries was set to 100, which is standard in DETR architectures and determines the maximum number of instances that the model can predict in a single image. This setting allows us to take into account class diversity and scenarios in which there are multiple instances of the same class in an image, e.g., atypical vessels. Unlike YOLO, where Optuna was used to search the hyperparameter space, RF-DETR was trained on a single, stable configuration tailored to the specific characteristics of the target classes. The tuning strategy was based on the configuration options available in both models. While YOLO allows for more extensive optimization of augmentation parameters, in the RF-DETR, augmentation is built-in and applied automatically; therefore, the training hyperparameters (such as batch size, number of epochs, and early stopping) were selected experimentally from among the available options.

Both models were trained on a computer running Ubuntu 22.04, using KFA2 GeForce^®^NVIDIA RTX 2080 Ti OC Black, 11GB GDDR6, 352-bit graphic card, manufactured by KFA2 in Dongguan, Guangdong, China in 2018.

Model evaluation was performed using the optimal weights, selected based on validation performance during training with the early stopping mechanism. The effectiveness of segmentation was assessed by comparing prediction masks with reference masks using four commonly used metrics [46,47]: Dice coefficient (1), IoU (2), precision (3), and sensitivity (4).

Dice = 2TP/(2TP + FP + FN)(1)

IoU = TP/(TP + FP + FN)(2)

Precision = TP/(TP + FP)(3)

Sensitivity = TP/(TP + FN)(4)

In Formulas (1)–(4), the values, TP (True Positive), FP (False Positive) and FN (False Negative) refer to the number of pixels in the segmentation areas. TP represents pixels that the model marked as an object and that actually belong to the object in the reference mask. FP represents pixels that the model incorrectly classified as belonging to the object, despite the fact that they are background in the reference mask, which happens in the oversegmentation. FN consists of pixels belonging to the object in the reference mask that the model failed to detect, classifying them as background instead; this typically occurs in the case of undersegmentation. In practical implementation, to prevent division by zero, a small constant value (1 × 10^−8^) was added to the denominators. These metrics allowed us to assess both the degree of coverage of anatomical area and findings and the model’s ability to detect them correctly.

## 3. Results

This section presents the results of experiments conducted using YOLO v11 and RF-DETR (RFDETRSegPreview) models. We focused on the image segmentation for selected classes. The aim of this part of the research is to compare the quality of segmentation obtained in both approaches. The metrics selected for analysis calculated on the same images allowed for the evaluation of predicted masks and a direct comparison of both models.

At the beginning, metrics calculated for test data determined for selected classes are presented. They were divided into anatomical elements: Squamous epithelium, Transformation zone, Cervix, External os; Medical instruments class; and findings: Polyps, Erythroplakia, Iodine-negative zone and Acetowhite epithelium. The number of images used for training, validation, and testing is presented in Table A4, Appendix A. As one can notice, the data sample size differs significantly between classes, which is particularly important in performance assessment, which constitutes a limitation of this study.

As one can notice, the dataset is not balanced. The most numerous classes describe key anatomical structures visible in colposcopic images (Cervix, External os, and Squamous epithelium). Some classes have significantly fewer examples, especially Medical instruments and colposcopic findings (Polyps are the least numerous class). Differences in the number of individual classes may affect the quality of the models.

### 3.1. Anatomical Structures

**Figure 1:**
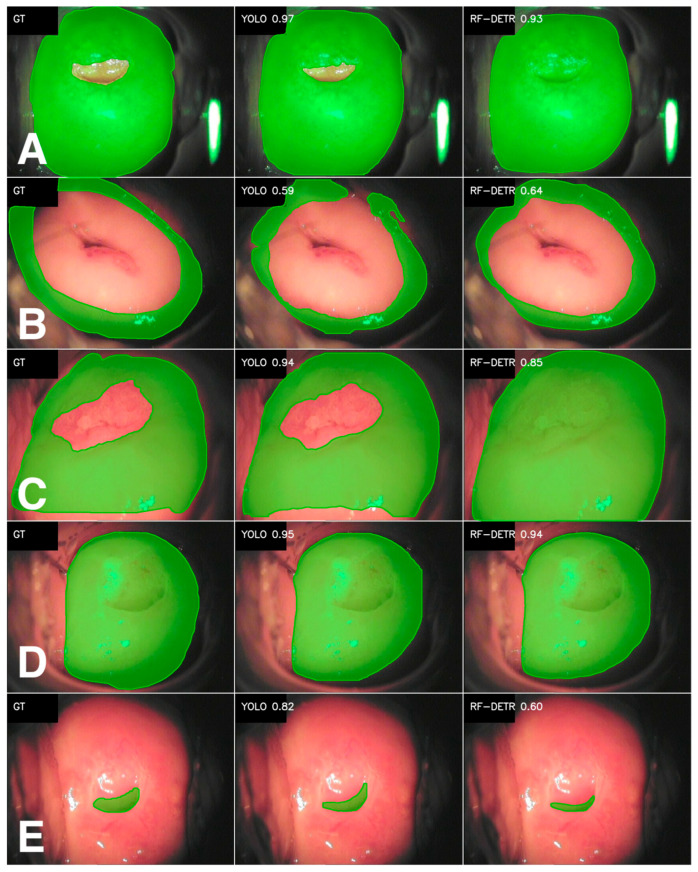
Segmentation results of YOLO v11 and RF-DETR models for anatomy classes. The figure presents a visual comparison of pixel-level segmentation performance for selected data examples. Sections A to E correspond to analyzed anatomical classes ((**A**)—Metaplastic squamous epithelium; (**B**)—Original squamous epithelium; (**C**)—Transformation zone; (**D**)—Cervix; (**E**)—External os). Each section is a triplet of images stacked horizontally, representing GT—ground truth (the manually annotated mask); YOLO—the segmentation predicted by the YOLO v11 model; and RF-DETR—the segmentation predicted by the RF-DETR model. The Dice metric for each presented prediction is imprinted on the corresponding image.

The segmentation results for both Metaplastic and Original squamous epithelium are given in Table 2. For the Metaplastic variant, both models achieve high mask quality, but YOLO performs better for each metric (Dice 0.87 vs. 0.81; IoU 0.80 vs. 0.74; precision 0.87 vs. 0.77; recall 0.92 vs. 0.89). It is important to mention that the median values for the Dice score are identical for both models (0.93), which may indicate problems with the RF-DETR model in the case of more difficult images, as confirmed by higher standard deviations (0.16 vs. 0.27). It was observed that the RF-DETR model has a tendency to cover the External os. The YOLO model does not have this problem. The effect of the sample segmentation with the Dice metric imprinted is shown in Figure 1A.

In the case of Original squamous epithelium, the metrics are significantly lower, regardless of the model (Table 2). This class has significantly fewer examples than Metaplastic squamous epithelium (training: 387 vs. 1536; test: 56 vs. 220). In this case, YOLO achieves slightly higher Dice and IoU metrics and precision but significantly lower recall (0.77 vs. 0.90). This means that RF-DETR more often marks the correct region but at the cost of a higher number of redundant pixels (lower precision). High standard deviations indicate high variation in segmentation difficulty, which is typical with a limited number of examples. In addition, for Original squamous epithelium, annotations made in CVAT were often confused with Metaplastic squamous epithelium. Sample predictions are shown in Figure 1B.

The results obtained for the Transformation zone class are high in both cases, and the differences between the models are not significant. YOLO achieves slightly better results for Dice and IoU (0.83 vs. 0.82 and 0.74 vs. 0.73) and also has higher precision (0.79 vs. 0.76). RF-DETR stands out with its very high sensitivity (recall 0.95 with a median of 0.99), which suggests that it misses fragments of the transition zone less frequently, even if this sometimes comes at the expense of boundary precision (decrease in precision compared to YOLO). RF-DETR scores achieved lower standard deviations for Dice, IoU, and recall, so its predictions are more consistent between consecutive images. YOLO, with lower recall but higher precision and IoU, seems to perform segmentation more carefully. An example segmentation for one of the test images is shown in Figure 1C.

The Cervix is the most numerous class among the analyzed classes. This is due to the idea of colposcopic images itself. For this class, both models achieve very high, comparable segmentation quality. What differs between the two models is the stability of the YOLO model, which has lower standard deviations across most metrics (especially recall, 0.04 vs. 0.11) and performs better even in more difficult cases. The Cervix usually occupies a large area of the colposcopy image, is usually clearly visible, and has a repeatable shape, which supports learning and explains the very good results for this class. An example of the segmentation effect is shown in Figure 1D.

For the External os class, the differences between the models are already much more significant in favor of YOLO. The Dice metric achieves higher average values of 0.66 (YOLO) vs. 0.53 (RF-DETR), similarly to IoU (0.54 vs. 0.43). The biggest difference in favor of YOLO can be observed in the recall metric (0.78 with a median of 0.86 vs. 0.53 with a median of 0.64). Analysis of the metrics suggests that RF-DETR more often misses parts of the External os or fails to locate it in clinically challenging cases. YOLO finds the correct image segment much more often and performs more consistently (lower standard deviations). It is worth mentioning that this result is not due to a lack of data—the External os is one of the most numerous classes (Table 1). The difference in quality compared to the similarly numerous Cervix class is rather due to the nature of the class itself—the External os is small, has a changing shape, is often covered, and its boundaries are much more difficult to detect than in the case of the Cervix. An example of the segmentation result for both models is shown in Figure 1E.

### 3.2. Medical Instruments

**Figure 2:**
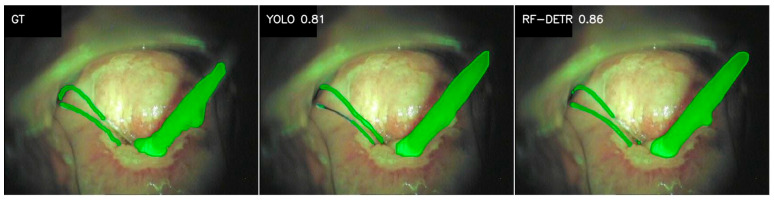
Segmentation results of YOLO v11 and RF-DETR models for the Medical instruments class. Image columns represent GT—ground truth (the manually annotated mask); YOLO—the segmentation predicted by the YOLO v11 model; and RF-DETR—the segmentation predicted by the RF-DETR model. The Dice metric for each presented prediction is imprinted on the corresponding image.

In addition to anatomical classes, we analyzed the class of Medical instruments used during colposcopy examinations including swabs, dressing forceps, vaginal speculum and IUD threads. The RF-DETR model performs better for this class. The average values of all metrics are noticeably higher for the RF-DETR model (Dice 0.73 vs. 0.62; IoU 0.64 vs. 0.54). However, the median metrics are quite high for both models, so in typical cases, the segmentation is rather correct, and the advantage of RF-DETR is determined by more difficult images, which is confirmed by the standard deviations. In YOLO, they are higher (e.g., Dice 0.38 vs. 0.3; IoU 0.35 vs. 0.29, recall 0.41 vs. 0.32), which indicates higher model variability. Medical instruments is a class in which the size of the set can have a significant impact on the interpretation of results (Table A4, Appendix A, 138/40/20). With such a data scale, any annotation error, tool type variety, or lighting conditions significantly affects the training process. In addition, instruments usually occupy a small part of the image and have thin edges, which is more difficult to segment than large anatomical structures such as the Cervix. The RF-DETR model showed greater tolerance to these difficulties, especially with a limited dataset. Sample segmentation results for the Medical instruments class are shown in Figure 2.

### 3.3. Colposcopic Findings

The first of the presented colposcopic findings is the Polyp class. In this case, the results are quite ambiguous—it all depends on whether we focus on averages or medians in the analysis. In terms of averages, RF-DETR performs better (Dice 0.70 vs. 0.64; IoU 0.61 vs. 0.59), and it also has higher precision and sensitivity. At the same time, the medians are very high for both models and even slightly better for YOLO (Dice 0.90 vs. 0.87; IoU 0.82 vs. 0.77; recall 0.92 vs. 0.90). Data analysis indicates that for less complex findings, both approaches segment polyps correctly. The differences in the mean originate from individual difficult images, where YOLO performs worse in terms of quality—similarly to the Medical instruments class. This is well illustrated by the standard deviations for individual metrics, which are markedly higher for the YOLO model.

**Figure 3:**
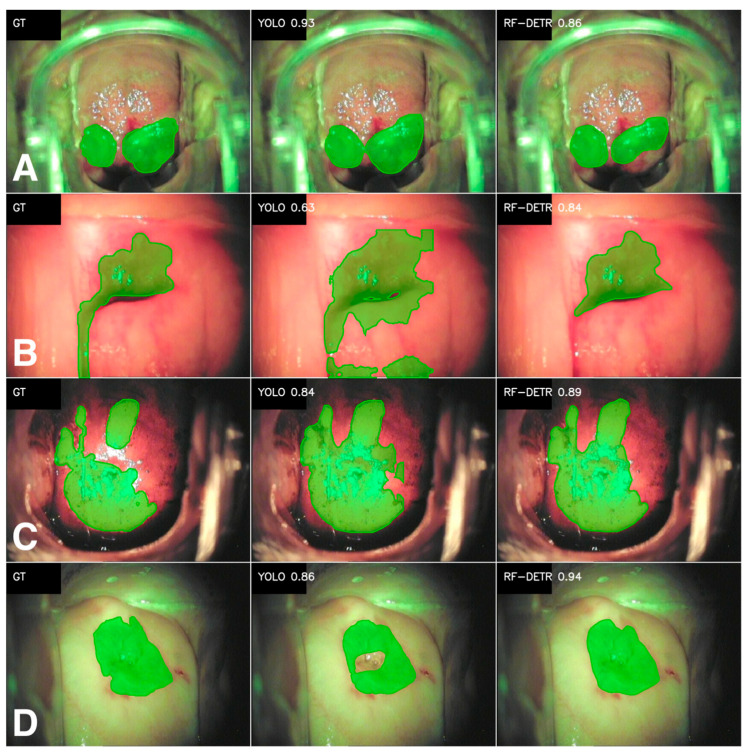
Segmentation results of YOLO v11 and RF-DETR models for anatomy classes. Sections A to D correspond to analyzed findings classes ((**A**)—Polyp, (**B**)—Erythroplakia, (**C**)—Iodine-negative zone, (**D**)—Acetowhite epithelium). Each section is a triplet of images stacked horizontally, representing GT—ground truth (the manually annotated mask); YOLO—the segmentation predicted by the YOLO v11 model; and RF-DETR—the segmentation predicted by the RF-DETR model. The Dice metric for each presented prediction is imprinted on the corresponding image.

The Polyp class is the least numerous class among those analyzed. With such a small dataset, individual failed predictions significantly affect the final averaged measures. The situation is analogous to that of the Medical instruments class, and a certain pattern can be observed. The RF-DETR model seems to segment better for small datasets. Examples of Polyp class segmentations are shown in Figure 3A.

The Erythroplakia class is another of the analyzed classes belonging to colposcopic findings. In terms of quantity, Erythroplakia has significantly fewer examples than the most representative anatomical structures (Table A4, Appendix A, 410/118/59). In the case of this class, the RF-DETR model achieves better results. The average values are higher for Dice (0.7 vs. 0.62) and IoU (0.6 vs. 0.52), as well as precision (0.71 vs. 0.63) and recall (0.74 vs. 0.68). In this case, the medians also indicate an advantage for the RF-DETR model (Dice 0.81 vs. 0.78; IoU 0.68 vs. 0.64; precision 0.86 vs. 0.80; recall 0.88 vs. 0.83). Nevertheless, the variance in results is still significant in both approaches (standard deviation 0.29–0.35). This is characteristic of classes with higher visual variability and less clear boundaries. Sample segmentation results for the Erythroplakia class are shown in Figure 3B.

Another analyzed finding is the Iodine-negative zone. Both models perform very similarly. The Dice, IoU, and recall metrics are almost identical (Dice: 0.79 for RF-DETR and 0.80 for YOLO; IoU: 0.71 vs. 0.72; recall: 0.83 vs. 0.86). The difference in precision is minimal in favor of RF-DETR. These results indicate that neither model holds a clear advantage when identifying the Iodine-negative zone. It is important to note the variance in results—the standard deviations are high (0.25–0.28) for all metrics, suggesting that the class is sometimes heterogeneous and may depend on the image taken or the coloring of the area. The medians are significantly higher than the means (e.g., Dice 0.91 vs. 0.89), so for most cases, the segmentation is correct, and the mean is lowered by individual difficult images (e.g., lower contrast). Sample segmentation results are shown in Figure 3C.

The last of the analyzed classes refers to the pathological lesion known as Acetowhite epithelium (Table 2, Figure 3D). It is the most numerous class related to colposcopy findings in the entire analyzed dataset. The average metric values indicate a clear advantage for RF-DETR (Dice 0.58 vs. 0.46, IoU 0.48 vs. 0.38, precision 0.61 vs. 0.49, and recall 0.66 vs. 0.51). The difference in favor of RF-DETR is also confirmed by the medians. In the case of the RF-DETR model, the median is noticeably higher than the average, suggesting that the model often segments areas correctly, but there are also more difficult examples that reduce the average result. The YOLO model more often makes mistakes in predicted masks and completely omits the prediction of some of them. However, the spread of results (standard deviations) is large for both models. This may be due to the specific nature of this pathology, namely blurred boundaries and varying appearance. The Acetowhite epithelium class is one of the most difficult pathologies to segment.

## 4. Discussion

The experiments showed that both YOLO v11n and RF-DETR allow for the prediction of segmentation masks for selected anatomical structures and findings in colposcopy images. However, the effectiveness of both models depends on the class and its specific features. It is important to mention that the aim of this study was to compare the analyzed architectures within a consistent data environment to minimize the impact of dataset variability on the results, rather than to conduct a full clinical validation or assess generalizability across different datasets.

The developed dataset contains classes that are highly imbalanced in terms of the number of instances. Some of them have very few examples, especially Medical instruments and Polyps, which have a direct negative impact on the variability of metrics (high standard deviations) and make the results highly sensitive to individual incorrect predictions. This imbalance is naturally connected to object frequency in clinical practice. While this distribution remains a challenge for model convergence, it ensures that the training dataset is representative of the real-world screening data. The classes also differ in terms of segmentation difficulty. Large and repeatable structures (such as the Cervix) are much easier to model than smaller ones with variable appearance and often covered, such as the External os or findings with blurred boundaries. Age-related anatomical variability may affect the visibility of certain cervical structures, particularly the transformation zone and the squamocolumnar junction (SCJ). Structures not visible in the image, such as a type 3 transformation zone with the new SCJ (n-SCJ) located within the cervical canal, were not annotated in the reference masks.

The study included three training configurations of YOLO models (Table A2, Appendix A) selected with the Optuna package. The configuration for each class was determined based on the model’s results for the validation set [48]. The most frequently used configuration was 3 (5/10 classes), followed by configuration 2 (3/10 classes), and the least frequently used was configuration 1 (2/10 classes). The more frequent selection of configuration 3 originates from the usage of two-stage training with layer freezing and moderate augmentation, which stabilizes segmentation. Configuration 2, with strong augmentation, was selected for Erythroplakia and Iodine-negative zone, among others, for which mask boundaries may be blurred. Configuration 1 was chosen because of its more cautious approach to augmentation, which was effective for the Acetowhite epithelium and External os classes. In the case of stronger augmentation, the mask boundaries for these classes were distorted, which negatively affected the quality of the models.

In several cases, the median metric is significantly higher than the mean (e.g., Metaplastic squamous epithelium (Dice metric median 0.93 with a mean of 0.81 for RF-DETR), which indicates correct segmentation of “typical” cases, but also the presence of “difficult” images that decrease the mean results.

The analysis of the results was divided into three groups of classes: anatomical structures, medical instruments, and findings for which a clear pattern of model behavior can be observed. For anatomical structures, both models achieve high scores, but the YOLO model more often dominates, distinguishing itself with better stability and repeatability of predictions. Good results are particularly relevant for numerous and clearly visible classes (Cervix, Metaplastic squamous epithelium, Transformation zone). The Original squamous epithelium class performed the worst in this group. This class is significantly less numerous than Metaplastic squamous epithelium, and because a number of misannotations were detected during the analyses, it is possible that there are still undetected errors in the dataset. Such errors can significantly reduce the performance of both models. As observed during the experiments, this type of problem cannot be solved by changing the model architecture. It would be necessary to improve the consistency of the labels.

In the case of the Medical instruments class, RF-DETR obtains better results. The Medical instruments class is a class with a small number of examples and high complexity. The objects are usually small, have thin edges and varied appearances, and are sometimes sensitive to light reflections. In such conditions, the YOLO model shows greater segmentation instability, while RF-DETR performs better in more difficult cases, as can be seen by comparing the average metric values with the median values.

The findings class group is distinguished by high complexity and variety of individual classes. The RF-DETR model achieves better results, but both models are able to correctly segment typical cases (high medians), and the differences mainly come from the results achieved on “difficult” images. The most difficult class to segment was Acetowhite epithelium, which is particularly complicated by blurred mask boundaries. In this case, the difference in favor of the RF-DETR model is more visible (means, medians), but the high standard deviations of the metrics indicate the need to increase the number of labels in the analyzed class in order to stabilize the prediction. This is clinically important, as dense acetowhite epithelium is associated with CIN2+ lesions, corresponding to HSIL in the current nomenclature. This finding manifests as a stain after acetic acid application as a thick, fast-appearing and long-lasting intensive white stain. However, the tissue reaction to acetic acid might also reveal a thin, transparent, white-ish stain that disappears quickly. This type of reaction suggests CIN-1 (LSIL) grade. The colposcopic examinations with photographic documentation on which the study was based were performed in accordance with current colposcopic standards (PTKiPSM protocols are listed in the Reference [8]). Each image was stored in the system along with the time of acquisition. Colposcopic assessment following the application of 3–5% acetic acid was performed continuously for at least 1 min, with particular attention paid to the dynamics of the appearance and persistence of acetowhite epithelium within the first 30–60 s after application. Similarly to acetowhite epithelium observed after the application of acetic acid, an iodine-negative zone in the iodine test (Schiller’s test) is also an important, albeit non-specific, clinical marker of possible high-grade lesions. Lack of iodine uptake may correspond to CIN2+ (HSIL) lesions, but it may also be observed in low-grade lesions.

In terms of implementation, in addition to prediction quality, model complexity is also important. YOLO v11n has approximately 2.88 million parameters, while RF-DETR has approximately 34.15 million. In practice, the YOLO model is lighter and easier to run in a clinical environment, allowing for quick predictions even with limited hardware resources while maintaining very good segmentation quality for many anatomical structures. Despite its greater complexity, RF-DETR shows potential in the segmentation of more difficult classes (Medical instruments, certain findings).

The results suggest that both models may be useful in future medical decision-making processes, but this will require further development and specific clinical validation. The segmentations made by the models can be used as suggestions. They can indicate areas to be assessed for pathology and facilitate the creation of medical documentation and the description of the colposcopy examination. The requirements for the meaningful use of models in clinical practice are high quality, consistency, and the largest possible number of annotations, because they directly affect the final training result and the subsequent quality of segmentation.

An important issue in the performed analysis is the problem of class imbalance, but one of the main limitations is the very small datasets for some of them. This makes it difficult to formulate clear conclusions about the superiority of one of the models. While class size inherently influences model performance, this study provides a baseline for future work on dataset expansion and targeted sample acquisition for underrepresented classes. Another limitation is the variability in image quality (lighting, coverage, unusable images) and the uncertainty of labels (Original squamous epithelium), which particularly affects the results obtained for “difficult” classes. It is worth noting that a single data division was used, which limits the generalizability of the conclusions, but the comparison remains reliable because both models were evaluated on an identical test set.

The approach we propose focuses on the segmentation of many different classes, including anatomical structures, medical instruments, and findings. The literature provides examples of the application of YOLO models in medicine for the detection and segmentation of various structures and pathologies, such as melanoma [49] or the detection and segmentation of polyps in colonoscopy [50]. Transformers, which have been gaining popularity in recent years, have also found wide application in the segmentation of medical images [51]. Nevertheless, it is difficult to find studies directly comparable to ours, especially those concerning the segmentation of many different classes in colposcopic images. A strong advantage of our approach is the use of our own unique dataset, which allows us to study multiple class groups (anatomical, findings, medical instruments), unlike most studies in the literature, which focus on the detection of the Cervix and its lesions.

The study [52] focuses on cervical segmentation using two different models, Mask R-CNN and MaskX R-CNN, on three combined datasets (CVT, ALTS, and Kaggle). Unlike the authors' approach, ours uses our own dataset, which is a definite advantage because it gives us full control and knowledge about how it was collected. Compared to the results of [52], the models we used achieved very similar Dice (0.951 vs. 0.947) and IoU (0.913 vs. 0.897) scores. It should be noted that the authors focused on only one anatomical class, while our approach covers many more diverse structures.

The article [53] proposes a two-step approach carried out similarly to our case on their own dataset. First, the Cervix was detected using Faster R-CNN, followed by segmentation of the Acetowhite epithelium. The results obtained by the authors (precision 0.74, dice 0.74) for Acetowhite epithelium segmentation exceed those achieved in our study, which shows that the two-step approach can be used in further research.

The article [54] presents a comparison of different methods for segmentation of the External os. The authors tested transformers (EndoViT/DPT), convolutional neural network-based solutions (SOTA CNN, DeepLabV3, YOLO v8, YOLO v11) and the PSPNet hybrid approach. The researchers worked with 913 images (200 cases) and obtained the best results for EndoViT/DPT (Dice = 0.5 and IoU = 0.39) and DeepLabV3 (Dice = 0.40 and IoU = 0.5). For comparison, segmentation of the External os in our study (YOLO v11) gave better Dice and IoU metrics (0.66 (0.73) ± 0.23, 0.54 (0.57) ± 0.23, Table 2). We used significantly more images to train the segmentation models (training 1505, validation 431, and testing 216). Although the selected metrics are higher in our case, it is important to note the significant impact of the size of the dataset, which probably helped to achieve better results.

It is easy to see that the study used only a part of the potential offered by the prepared dataset. Additional analyses using classes not analyzed here and their extensions by attributes will be studied in subsequent experiments. However, to provide clinical usability and prepare for implementation of live computer-aided analysis of colposcopy images [55], we also developed a web-based application prototype. The application is intended for use during a patient’s visit. It allows a safe transfer of images from colposcopic equipment to the object database so they can be assigned to the specific patient in the system. In a web user interface, the clinician can browse images and segmentation masks predicted for each of them. To enhance the creation of a printable colposcopy report, the clinician can add comments to every individual image. All input and inferred data are saved in a database for future reference.

Further work will also focus on enhancing the dataset, because this seems to be the main limitation, rather than the choice of model architecture itself [56]. We are going to focus on analyzing classes that are important for recognizing pathologies, such as Acetowhite epithelium, Mosaicism, Punctation, Atypical vessels and Erosions [57]. To achieve this, we intend to expand the dataset by increasing the number of annotations, especially for classes with low frequency (colposcopy findings, medical instruments), while ensuring consistency of labels. An important stage of further work will also be failure analysis conducted for the most challenging images and the expansion of the training set with such examples.

Further work could also include evaluating models on other data splits (e.g., using k-fold cross-validation) or on an independent dataset obtained under different acquisition conditions [56]. A hybrid approach could also be considered, in which the YOLO model would be used as a fast and stable anatomy segmentation model, and RF-DETR would be applied to more difficult classes. Implementing this type of approach is relatively simple due to the adopted training paradigm, in which each class is handled by a separate, independently trained model. The authors of the publication [58] compared two approaches to segmentation: the multiclass approach (one model for multiple classes) and a separate model for each class. The results show that the multiclass approach proved to be more effective. For MRI, it resulted in an increase in Dice metrics (from 0.73 to 0.86), IoU (from 0.79 to 0.85) and Dice for EFI (from 0.45 to 0.57). It is important to mention that in our case, we have a very large class imbalance, as well as internal similarity. Experiments conducted using a multiclass approach did not give satisfying results in our case. In this situation, the multiclass approach may face difficulties during training, as well as in distinguishing between classes with similar characteristics. A reasonable approach would be to increase the dataset and test the approach proposed in the publication [58] again. It is important to remember that the analyzed dataset consists of real-life images and therefore contains a typical representation of clinical cases and their real distribution. As a result, simply increasing the size of the dataset may not lead to an equal number of classes, and further work should also include the use of imbalanced learning methods. Due to these limitations, we decided to use separate models for each class in this study.

## 5. Conclusions

The conducted research concerned the segmentation of anatomical structures, medical instruments, and findings in colposcopic images using the YOLO v11 and RF-DETR models. The performed analysis allows us to conclude that both segmentation models enable the prediction of useful masks, and the quality of prediction depends on the characteristics and size of individual classes.

The most representative results were obtained for anatomical classes concerning large, clearly visible structures such as the Cervix or Metaplastic squamous epithelium. The most problematic were structures for which the amount of data was limited, which were small and had blurred boundaries, e.g., certain findings. In some cases, a clear difference was observed between the medians and the mean values of the metrics. This indicates correct segmentation of typical cases and a noticeable impact of “difficult” cases on the final averaged results.

The analyzed dataset was characterized by a strong class imbalance, which results from the very nature of real-life data. Some classes, such as Medical instruments or certain findings (e.g., Polyps), rarely appear in images. This leads to high metric variability and sensitivity to individual incorrect masks predicted by the models and therefore to the final segmentation quality assessment and comparison.

Analysis of the results in selected class groups shows (Table 2) that the YOLO model more often provides better stability for anatomical structures, while RF-DETR shows an advantage for more challenging classes, such as Medical instruments, and some findings. Considering implementation, the YOLO model is significantly lighter than RF-DETR (2.88 million vs. 34.15 million parameters) and would be better for anatomical structure segmentation. On the other hand, RF-DETR could be used instead of YOLO for segmentation of selected colposcopic findings.

The main limitations include class imbalance, differences in the quality of individual colposcopy images, and the issue of label inconsistency (especially Original squamous epithelium). Improvements to a large part of the dataset have allowed us to obtain satisfying results for the presented classes. In order to prepare further diagnostically relevant classes, we will continue to improve the dataset, focusing on increasing the consistency of annotations and expanding it to include underrepresented cases. Due to the typical diversity of clinical real-life data, simply increasing the size of the dataset may not improve class balance. It would also be beneficial to incorporate imbalanced learning techniques and explore alternative data partitioning methods to enhance model generalization.

## Figures and Tables

**Table 1:** Distribution of categorical classification tags and segmentation masks across the dataset. The counts include every occurrence of a class instance, regardless of specific attribute assignments.

Count	Class	Category	Annotation Type
151	Stage 0—before rinsing	Procedural stages	Tags
1794	Stage 1—after saline application		
1817	Stage 2—after application of acetic acid		
986	Stage 3—after application of Lugol’s solution		
1082	Normal colposcopic findings	Clinical assessment	
1260	Abnormal colposcopic findings		
2395	High quality image	Image quality	
1594	Low quality image		
959	Unusable image		
1956	Green filter	Technical parameters	
4461	Cervix	Physiological and anatomical	Masks
4214	Squamous epithelium		
3794	External os		
1059	Columnar epithelium		
829	nSCJ		
777	Transformation zone		
1169	Acetowhite epithelium	Findings	
1088	Erythroplakia		
699	Iodine-negative zone		
289	Rimmed glandular openings		
207	Endometriosis		
207	Mosaicism		
205	Polyp		
169	Punctation		
153	Atypical vessels		
102	Inflammatory changes		
96	Atrophy		
83	Exophytic changes		
75	Glandular ectopy		
38	Irregular surface		
38	Papilloma		
27	Leukoplakia		
8	Erosion		
6	Decidual changes (in pregnancy)		
4	Condyloma		
1850	Mucus	Obstacles and artifacts	
583	Blood		
282	Medical instruments		

**Table 2:** Segmentation metrics of trained models obtained on test data. Values are presented in the form of mean (median) ± SD.

RF-DETR				YOLO11n				Model
Recall	Precision	IoU	Dice	Recall	Precision	IoU	Dice	Metric
0.89 (0.98) ± 0.26	0.77 (0.90) ± 0.29	0.74 (0.87) ± 0.28	0.81 (0.93) ± 0.27	0.92 (0.96) ± 0.13	0.87 (0.93) ± 0.18	0.80 (0.88) ± 0.19	0.87 (0.93) ± 0.16	Squamous epithelium class (metaplastic)
0.77 (0.90) ± 0.31	0.42 (0.35) ± 0.30	0.37 (0.33) ± 0.26	0.48 (0.50) ± 0.29	0.58 (0.77) ± 0.37	0.49 (0.60) ± 0.33	0.39 (0.43) ± 0.28	0.50 (0.60) ± 0.32	Squamous epithelium class (original)
0.95 (0.99) ± 0.09	0.76 (0.79) ± 0.20	0.73 (0.78) ± 0.19	0.82 (0.88) ± 0.16	0.90 (0.95) ± 0.16	0.79 (0.89) ± 0.23	0.74 (0.82) ± 0.22	0.83 (0.90) ± 0.20	Transformation zone
0.95 (0.98) ± 0.11	0.92 (0.98) ± 0.17	0.89 (0.93) ± 0.16	0.93 (0.96) ± 0.15	0.97 (0.98) ± 0.04	0.94 (0.97) ± 0.10	0.91 (0.94) ± 0.10	0.95 (0.97) ± 0.07	Cervix
0.53 (0.64) ± 0.36	0.59 (0.76) ± 0.40	0.43 (0.51) ± 0.30	0.53 (0.67) ± 0.34	0.78 (0.86) ± 0.24	0.64 (0.71) ± 0.27	0.54 (0.57) ± 0.23	0.66 (0.73) ± 0.23	External os
0.75 (0.91) ± 0.32	0.76 (0.86) ± 0.30	0.64 (0.74) ± 0.29	0.73 (0.85) ± 0.30	0.67 (0.86) ± 0.41	0.59 (0.75) ± 0.38	0.54 (0.68) ± 0.35	0.62 (0.81) ± 0.38	Medical instruments
0.77 (0.90) ± 0.34	0.72 (0.88) ± 0.30	0.61 (0.77) ± 0.31	0.70 (0.87) ± 0.32	0.67 (0.92) ± 0.44	0.63 (0.88) ± 0.42	0.59 (0.82) ± 0.39	0.64 (0.90) ± 0.42	Polyp
0.74 (0.88) ± 0.33	0.71 (0.86) ± 0.31	0.60 (0.68) ± 0.29	0.70 (0.81) ± 0.31	0.68 (0.83) ± 0.34	0.63 (0.80) ± 0.35	0.52 (0.64) ± 0.31	0.62 (0.78) ± 0.33	Erythroplakia
0.83 (0.95) ± 0.28	0.80 (0.92) ± 0.28	0.71 (0.83) ± 0.28	0.79 (0.91) ± 0.27	0.86 (0.96) ± 0.25	0.78 (0.90) ± 0.28	0.72 (0.79) ± 0.26	0.80 (0.89) ± 0.25	Iodine-negative zone
0.66 (0.80) ± 0.33	0.61 (0.78) ± 0.37	0.48 (0.51) ± 0.32	0.58 (0.68) ± 0.33	0.51 (0.65) ± 0.40	0.49 (0.65) ± 0.41	0.38 (0.32) ± 0.34	0.46 (0.48) ± 0.38	Acetowhite epithelium

## Data Availability

The dataset created for this study cannot be shared due to their sensitive nature and data protection policies at the University Center for Women’s and Newborn’s Health.

## Appendix A

**Table A1:** Summary of the most important features of the models used for training.

RF-DETR	YOLO v11	Feature
Transformer, DETR detector type, built on a DINOv2 vision transformer backbone	one-stage CNN	Model type
COCO dataset	COCO dataset	Pretraining
34.15 million	2.88 million	Number of parameters
COCO: JSON files (paths to images, annotations, categories)	YOLO: .txt files (class + polygon), YAML (paths)	Input data format
2025	2024	Year of introduction

**Table A2:** Configuration parameters for YOLO training hyperparameter optimization using Optuna software.

Configuration 3	Configuration 2	Configuration 1	Feature
Moderate: hsv_h = 0.04 degrees = 6.0 translate = 0.08	Strong: hsv_h = 0.07 degrees = 10.0 mosaic = 1.0 mixup = 0.15	Moderate: hsv_h = 0.02 degrees = 5.0 translate = 0.05 scale = 0.10	Augmentation
AdamW	SGD	AdamW	Optimizer
6 × 10^−4^	0.01	5 × 10^−4^	Start learning-rate
200	300	200	Epochs
10	0	0	Freeze
50	50	25	Patience

**Table A3:** Main parameters of the RF-DETR model and its training.

Value	Parameter
RFDETRSegPreview	Model
350	Epochs
8 (batch = 2 × accum = 4)	Effective batch
0.0001	LR
Yes patience = 15 min_delta = 0.001	Early stopping
num_queries = 100 num_select = 100 amp = False eval_interval = 1 save_best = True num_workers = 0	Key attributes

**Table A4:** Number of images in the dataset for individual classes.

Optuna Configuration	Test Set	Validation Set	Training Set	Class
3	56	111	387	Squamous epithelium (original)
3	220	440	1536	Squamous epithelium (metaplastic)
2	245	490	1713	Cervix
1	216	431	1505	External os
3	54	107	374	Transformation zone
3	20	40	138	Medical instruments
3	14	27	94	Polyp
2	59	118	410	Erythroplakia
2	48	96	331	Iodine-negative zone
1	78	155	542	Acetowhite epithelium
